# A systematic review on self-management education campaigns for back pain

**DOI:** 10.4102/sajp.v75i1.1314

**Published:** 2019-08-13

**Authors:** Loveness A. Nkhata, Yolandi Brink, Dawn Ernstzen, Quinnette A. Louw

**Affiliations:** 1 Division of Physiotherapy, Department of Health and Rehabilitation Sciences, Stellenbosch University, Cape Town, South Africa; 2 Department of Physiotherapy, School of Health Sciences, University of Zambia, Lusaka, Zambia

**Keywords:** back pain, self-management, education, media, campaign

## Abstract

**Background:**

Evidence-based clinical practice guidelines on back pain recommend early management and use of approaches that emphasise self-management, psychological and physical therapies. Lately, mass media campaigns, addressing misconceptions about back pain, have been conducted in developed countries.

**Objectives:**

This study retrieved and synthesised the contents of back pain messages and described the outcomes and effectiveness of the media campaigns.

**Method:**

Seventeen key words and 10 electronic databases were used to conduct a search between February and July 2018. Authors screened titles, abstracts and full-text articles independently to identify eligible studies. Data were reported using narratives because of heterogeneity in the outcomes.

**Results:**

Appraisal of articles was done using the Physiotherapy Evidence Database scale for randomised controlled trials (RCT) (one) or the Joanna Briggs Institute checklist for non-RCT (four). The campaigns were conducted in the general population in Australia, Canada, Norway, the Netherlands and Scotland. The message ‘stay as active as possible’ increased participants’ awareness and influenced their health beliefs and healthcare utilisation behaviours resulting in reductions in sick leave days, work disability, healthcare utilisation and claims.

**Conclusion:**

The back pain campaign message ‘stay as active as possible’ increased participants’ awareness and influenced their health beliefs and healthcare utilisation behaviours. Even though the campaigns were done in high-income countries, their contents and methods are transferable to developing countries. However, their implementation must be tailored and efficient and cost-effective methods need to be explored.

**Clinical implications:**

Providing information on back pain can contribute to significant changes in sickness behaviours and beliefs.

## Introduction

Back pain is a global health challenge and a leading common condition that causes disability and affects especially the working population worldwide (Forster et al. 2018; Hartvigsen, Natvig & Ferreira 2018; Hoy et al. 2010).

Globally, approximately 149 million workdays at a cost of US$100–200 billion are lost because of back pain yearly (Vos et al. 2012). Even though most episodes of back pain recover within a few weeks, most individuals seek care from health institutions which results in an economic burden for both the healthcare systems and the affected individuals (Forster et al. 2018; Hartvigsen et al. 2018; Hoy et al. 2010; Montegomery et al. 2017; Morris et al. 2018). Several strategies such as ergonomic training, environmental engineering, use of devices or equipment and exercise therapy or physiotherapy are used to manage back pain because the aetiology is multifactorial (Friemann et al., 2015; Jaromi et al. 2012; Soon-Lae & Jong-Eun 2010). Nonetheless, evidence-based clinical practice guidelines (Michaleff et al. 2014; Stochkendahl et al. 2018; Qaseem et al. 2017) on back pain recommend early management and use of biopsychosocial active approaches such as back pain media campaigns that promote self-management and functional improvement (Buchbinder et al. 2018; Forster et al. 2018; Hoy et al. 2010). Media campaigns are a health strategy used to deliver health messages to the community (Buchbinder et al. 2008). In addition, they influence population attitudes, beliefs and change in health risk behaviours (Buchbinder et al. 2008). In healthcare, back pain media campaigns address pain coping strategies and biomedical factors using simple evidence-based messages (Buchbinder et al. 2018; Forster et al. 2018; Hoy et al. 2010), including back pain not being a severe problem, disability from back pain being able to be improved and prevented by positive attitudes and that there is a lot that one could do to help one self (Buchbinder et al. 2008). Notably, these campaigns have not yet been conducted in low- and middle-income countries (LMICs), but have been done in high-income countries (HICs) among the general population with remarkable success in shifting back pain beliefs, decline in worker compensation claims and reduced healthcare utilisation because of back pain (Forster et al. 2018; Hoy et al. 2010; Waddell et al. 2007; Werner et al. 2008). As a result, recommendations have been made for these campaigns to be contextualised and conducted in specific populations (Buchbinder et al. 2018; Forster et al. 2018; Hoy et al. 2010). This is because tailored campaigns promise to be an effective and affordable strategy in mitigating the effects and burden of back pain (Forster et al. 2018). These campaigns seemingly are a promising method for promoting back care in Africa and other developing regions where the projected increase in back pain disability has a negative impact on societal, economical and public health issues. The purpose of this review was to retrieve and synthesise the content of back pain campaigns and describe the outcomes and effectiveness of the campaigns.

## Methodology

The preferred reporting items for systematic reviews and meta-analyses (PRISMA) guidelines were used in defining the participants, interventions, comparisons, outcomes and study designs (PICOS).

### Eligibility criteria

Intervention studies such as randomised controlled trials (RCTs), quasi-experimental case–control, crossover trials and observational studies published in English were considered for this review. The population of interest was the general public and the intervention was back pain educational campaigns. Comparisons, such as controls not exposed to the intervention, were also considered and outcomes included process and measures such as pain, participant activation measure, number of sick leave days, back pain beliefs measure, frequency of doctor visits and frequency and amount of pain relief medication.

### Information sources and search strategy

Using the MEDLINE search strategy, the Cochrane Occupational Safety and Health database, MEDLINE, EMBASE, SCOPUS, Physiotherapy Evidence Database (PEDro), the National Institute for Occupational Safety and Health (NIOSH) database and the International Occupational Safety and Health Information Centre were searched. The search was conducted between October 2017 and March 2018 and it included articles from 1990 to 2018. The search terms included ‘educational’, ‘interventions’, ‘campaigns’, ‘treatment’, ‘self-management’, ‘musculoskeletal pain’, ‘back pain’, ‘BP’, ‘low back’, ‘lower back’, ‘LBP’, ‘pain’, ‘injuries’, ‘management’, and ‘nurse’, ‘nurses’ and ‘nursing’. The authors obtained and screened titles, abstracts and citations identified by the searches and then retrieved full-text articles independently to identify eligible studies published in English for independent selection. In addition, hand searching of relevant journals, bibliographic databases, dissertations and direct communication with authors of included studies was done to obtain clarity. Other resources were reference lists of relevant articles and registers of clinical trials, including the World Health Organisation International Clinical Trials Registry Platform.

### Data extraction and analysis

Authors independently performed data extraction on contents of back pain campaign messages from selected articles taking into consideration checks for discrepancies and processing which were resolved by consensus (Higgins et al. 2011). Contents which were retrieved included study method, objectives, participants, intervention type, outcome measures, results, references, intervention messages, mode of transmission and duration. The results from the articles were described descriptively because of heterogeneity.

### Methodological appraisal and assessment of risk of bias

Appraisal of the methodology for RCTs was done using the PEDro scale (Verhagen et al. 1998) which assesses external validity (criterion 1), internal validity (criteria 2–8) and statistical accuracy (criteria 9–10). In addition, the scale contains 11 items, scored as Yes/No, which is either present (1) or absent (0). Non-RCTs were assessed using the Joanna Briggs Institute (JBI) appraisal (Tafanaru et al. 2015) which has items that are scored as Yes/No. For risk of bias, the Cochrane risk of bias in non-randomised studies of interventions (ROBINS-I) tool was used (Higgins et al. 2016; Thompson et al. 2018; Sterne et al. 2016). This tool focuses on assessing internal validity using seven specific bias domains which include confounding, selection of participants, classification of interventions, missing data, measurements of outcomes and selection of reported results (Sterne et al. 2016; Thompson et al. 2018). In addition, it contains question items measured on a Likert scale of ‘yes’ for minimal risk of bias, ‘probably yes’, ‘probably no’ and ‘no’ for elevated risk of bias (Higgins et al. 2016; Sterne et al. 2016; Thompson et al. 2018). These include the following: is there potential for confounding of the effect of intervention in this study; was selection of participants into the study based on participant characteristics observed after the start of intervention; were intervention groups clearly defined; were there deviations from the intended intervention beyond what would be expected in usual practice; were outcome data available for all, or nearly all, participants; could the outcome measure have been influenced by knowledge of the intervention received and is the reported effect estimate likely to be selected, based on the results, from multiple outcome measurements, analyses of the intervention or different subgroups?

### Ethical considerations

This review is part of the Project ID:7431 about the effectiveness of a contextualised back pain campaign for nurses in Lusaka, Zambia. Ethical clearance for the project was sought from the Stellenbosch University Health Research Ethics Committee (SU-HREC) – Project ID: 7431 and HREC Reference #: S18/06/125.

## Results

### Description of studies

**FIGURE 1:**
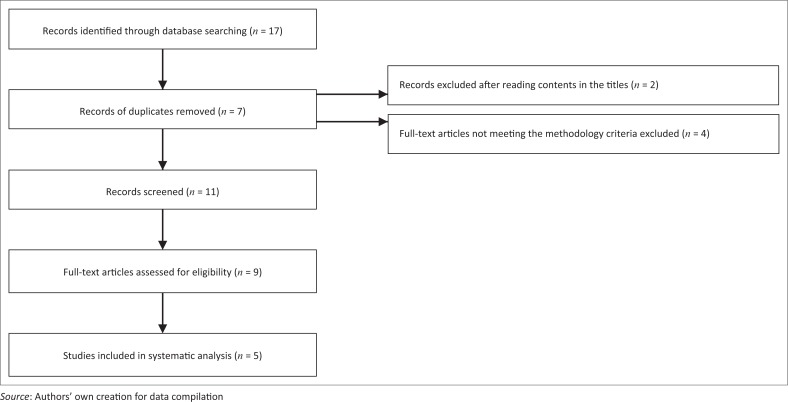
Article selection process using the PRISMA flow chart.

Following electronic searching, 17 potentially relevant articles were identified. Titles, keywords and abstracts of these articles were assessed, and 11 eligible articles were selected and publications obtained. From the 11 eligible articles, five studies were included in our review. Figure 1 illustrates the article selection process.

### Methodological appraisal

Appraisal of the methodology for Suman et al. (2017) was done using the PEDro scale (Verhagen et al. 1998) and a score of 6/11 was obtained. For the remaining four articles (Buchbinder et al. 2008; Gross et al. 2010; Waddell et al. 2007; Werner et al. 2007), the JBI appraisal (Tafanaru et al. 2015) was used and items were scored as Yes/No, which is either present (1) or absent (0). The overall score for the four articles was 8/11; details on the appraisal scores for included studies are shown in Tables 1 and 2.

**TABLE 1:** Evidence grading scores according to Physiotherapy Evidence Database criteria.

Author	Item 1	Item 2	Item 3	Item 4	Item 5	Item 6	Item 7	Item 8	Item 9	Item 10	Item 11	Score
Suman et al. (2017)	Y	UC	UC	Y	UC	UC	UC	Y	Y	Y	Y	6/11

*Source*: Authors’ own creation for data compilation

Options for signalling questions: Yes (Y), No (N), Unclear (UC), Not applicable (NA).

Items refer, (1) Eligibility criteria were specified; (2) Participants were randomly allocated to groups (in a crossover study, subjects were randomly allocated in order in which treatments were received; (3) Allocation was concealed; (4) The groups were similar at baseline regarding the most important prognostic indicators; (5) There was a blinding of all participants; (6) There was a blinding of all therapists who administered the therapy; (7) There was blinding of all assessors who measured at least one key outcome. (8) Measures of at least one key outcome were obtained from more than 85% of the participants initially allocated to groups. (9) All participants for outcome measures were available and received the treatment or control condition as allocated or, where this was not the case, data for at least one key outcome were analysed by `intention to treat’; (10) The results of between-group statistical comparisons are reported for at least one key outcome; (11) The study provides both point measures and measures of variability for at least one key outcome.

**TABLE 2:** Evidence appraisal according to the JBI appraisal checklist for quasi-experimental studies.

Author	Item 1	Item 2	Item 3	Item 4	Item 5	Item 6	Item 7	Item 8	Item 9	Score	Overall appraisal
Gross et al. (2010)	Y	N	Y	Y	Y	Y	Y	Y	Y	8/9	Included
Buchbinder et al. (2008)	Y	N	Y	Y	Y	Y	Y	Y	Y	8/9	Included
Waddell et al. (2007)	Y	N	Y	Y	Y	Y	Y	Y	Y	8/9	Included
Werner et al. (2007)	Y	N	Y	Y	Y	Y	Y	Y	Y	8/9	Included

*Source*: Authors’ own creation for data compilation

Options for signalling questions: Yes (Y), No (N), Unclear (UC), Not applicable (NA).

Items refer, (1) Is it clear in the study what is the cause and what is the effect (i.e. there is no confusion about which variable comes first)?; (2) Were the participants included in any comparisons similar?; (3) Were the participants included in any comparisons receiving similar treatment or care, other than the exposure or intervention of interest?; (4) Was there a control group?; (5) Were there multiple measurements of the outcome both pre and post the intervention or exposure?; (6) Was follow-up complete and if not, were differences between groups in terms of their follow-up adequately described and analysed?; (7) Were the outcomes of participants included in any comparisons measured in the same way?; (8) Were outcomes measured in a reliable way?; (9) Was appropriate statistical analysis used?

### Study sample description

Five full-text reports (Buchbinder et al. 2008; Gross et al. 2010; Suman et al. 2017; Waddell et al. 2007; Werner et al. 2007) on self-management education campaigns of back pain were included in our review (Table 3). Notably, these campaigns were conducted in HICs among the general population in Australia (Buchbinder et al. 2008), Canada (Gross et al. 2010), Norway (Werner et al. 2007), the Netherlands (Suman et al. 2017) and Scotland (Waddell et al. 2007). Methodological designs of the studies included quasi-experimental (Buchbinder et al. 2008; Gross et al. 2010; Werner et al. 2007) before and after observational study (Waddell et al. 2007) and a mixed-methods step-wedge RCT (Suman et al. 2017).

**TABLE 3:** Characteristics of included studies.

Author	Campaign	Objective	Study design
Suman et al. (2017)	eHealth media campaign	Evaluated process of a multimedia campaign to improve back beliefs in patients with non-specific low back pain (LBP)	Mixed methods step-wedge RCT
Gross et al. (2010)	Back @ it	Evaluated a back pain mass media campaign’s impact on population back pain beliefs, work disability and health utilisation outcomes	Quasi-experimental
Buchbinder et al. (2008)	Back Pain: Don’t Take It Lying Down	Aimed at shifting the responsibility of control onto the individual and promoting self-management	Quasi-experimental
Waddell et al. (2007)	Working Backs Scotland	Aimed at changing public beliefs about the management of back pain	Before–after observational study
Werner et al. (2007)	Active back project	Evaluated the effect of a media campaign on popular beliefs about LBP and eventual changes in sick leave, imaging examination and surgery	Quasi-experimental

*Source*: Authors’ own creation for data compilation

RCT, randomised controlled trial.

### Study interventions content

The back pain media campaign contents on the intervention’s messages, mode of transmission and duration for the campaigns are shown in Table 4. Campaign messages were different in the campaigns, but a few messages were similar in some campaigns such as ‘don’t take it lying down’ (Buchbinder et al. 2008; Gross et al. 2010) and ‘stay active’ were common in the campaigns (Buchbinder et al. 2008; Gross et al. 2010; Suman et al. 2017; Waddell et al. 2007; Werner et al. 2007). Campaign messages were transmitted using television (Buchbinder et al. 2008; Gross et al. 2010; Werner et al. 2007), radio (Buchbinder et al. 2008; Gross et al. 2010; Waddell et al. 2007), bill boards (Buchbinder et al. 2008; Gross et al. 2010; Waddell et al. 2007), workshops (Buchbinder et al. 2008; Gross et al. 2010), celebrities (Buchbinder et al. 2008), newspaper articles (Werner et al. 2007), websites (Gross et al. 2010; Suman et al. 2017; Waddell et al. 2007) and flyers (Buchbinder et al. 2008; Gross et al. 2010; Suman et al. 2017; Waddell et al. 2007; Werner et al. 2007). Duration and follow-up period for four campaigns were 3 years.

**TABLE 4:** Interventions, mode of transmission and duration.

Authors	Suman et al. (2017)	Gross et al. (2010)	Buchbinder et al. (2008)	Waddell et al. (2007)	Werner et al. (2007)
Back pain messages	Stay active, Continuing or returning to work, Coping with back pain.	Back pain: don’t take it lying down The key to feeling better sooner is to stay active	Back pain is not a serious problem continue usual activities Don’t rest for prolonged periods Continue exercising and remain at work if possible Positive attitudes are important, and it is up to you X-rays are not useful Surgery may not be the answer to keep employees at work	Stay active Try simple pain relief If you need it, get advice Don’t take back pain lying down There’s a lot you can do to help yourself The prognosis is usually good	Back pain is rarely caused by dangerous illness X-ray rarely reveals the cause of back pain A back in motion improves faster Work with your back, one recovers faster by returning to work as soon as possible Only a few people with back pain need surgery
Mode of transmission	Website, e-videos and pamphlets	Website, radio, bus adverts, posters, pamphlets, billboard, articles in public or industry news publications and TV public service announcements	TV, radio and printed adverts; outdoor billboards, posters, seminars, workplace visits and publicity articles	Website, radio and printed adverts; billboards, posters, seminars, workplace visits and publicity articles	Website, TV, radio and cinema adverts; posters with the campaign messages at health clinics
Duration	2 years (2010–2012)	3 years (2005–2008)	3 years (1997–1999)	3 years (2000–2003)	3 years (2002–2005)

*Source*: Authors’ own creation for data compilation

### Assessment of outcomes

*Outcomes and overall effect of the campaigns on awareness, participant activation and satisfaction*: Outcomes that were measured in the campaigns are summarised in Table 5. A significant improvement in back pain beliefs in the general population was observed in the Australia (*F* = 7.43; *p* < 0.001), Canada (565% – 63%), Norway (21.2% – 22.6%) and Scotland (*p* < 0.001) campaigns (Buchbinder et al. 2008; Gross et al. 2010; Waddell et al. 2007; Werner et al. 2007). However, authors in Canada indicate that although positive outcomes were observed, there was no meaningful statistical significance (*p* = 0.13) on the overall effect of the campaign (Gross et al. 2010).

**TABLE 5:** Outcomes and the effectiveness of the campaigns.

Author	Campaign awareness	Population back pain beliefs and staying active	Patient satisfaction	Sick leave, healthcare utilisation and imagining use	Medical claims and incidence of claims	Overall effect of campaign
Suman et al. (2017)	Awareness increased with time	Proportions not reported	Satisfaction increased with use	Not reported	Outside study scope	Patient satisfaction increased use of media campaign platform
Gross et al. (2010)	49.2% (Treatment), 38.8% (controls)	Back pain beliefs 56% – 63% (*p* = 0.13) Staying active *p* = 0.008	Outside study scope	Healthcare utilisation reduced	13% Reduction	Proportion of subjects agreeing to stay active increased from 56% to 63% (*p* = 0.008). But no statistically significant effects were seen in sick leave outcomes
Waddell et al. (2007)	60%	(*p* < 0.001) Significant reversal in back pain beliefs	Outside study scope	11% Downward trends were observed	Fewer spells days of back pain. No new awards of social security benefits for back disorders	Significant, shift in public beliefs about staying active, 5.5% – 15.7%, *p* < 0.001), but no effect on sickness absence, no new awards of social security benefits for back pain
Werner et al. (2007)	29% – 39% *p* = 0.000	Staying active increased from 21.2% to 22.6%	Outside study scope	13% Reduction on sickness leave days; reduced X-rays use 35% (intervention), 33% (control)	Observed increase in surgery rate claims in both intervention and control	Significant shift in low back pain beliefs in general population, importance of remaining active and at work. Reduced use of X-rays
Buchbinder et al. (2001)	47% – 86%	Staying active 1.9 (CI 1.3–2.5) before to 3.2 (CI 2.6–23.9) (*F* = 7.43; *p* < 0.001)	Outside study scope	15% (Controls) and 20% (intervention) Reduced use of X-rays	Claims reduced by 15% *p* = 0.013	Significant, shift in population low back pain beliefs, behaviour and reduction in workers’ medical compensation claims

*Source*: Authors’ own creation for data compilation

CI, confidence interval.

*Back beliefs measure and reported change in back beliefs following the campaigns*: The back beliefs questionnaire was used to measure back pain beliefs (Buchbinder et al. 2008; Waddell et al. 2007; Werner et al. 2007). Significant improvements 1.9–3.2 (confidence interval [CI] 1.3–2.5 to 2.6–23.9) in population back beliefs in Australia were observed and sustained even 3 years after the campaign (Buchbinder et al. 2008). In addition, a satisfactory significant reversal (CI 21.2–22.6) in the balance of back beliefs was reported in Scotland (Waddell et al. 2007) while in Canada it was 56% – 63% (Gross et al. 2010).

*Health utilisation, back claims and number of sick leave days following the campaigns*: The ability to self-manage and better use of X-rays was reported (Buchbinder et al. 2008; Werner et al. 2007). Sick leave days and number of claims for back problems declined over the campaign duration by 5% (Buchbinder et al. 2008). Furthermore, a general decline in the number of sickness days and overall reduction 5% (*p* = 0.013) in claims were observed (Buchbinder et al. 2008). In addition, a generally downward trend was observed: 13% reduction in the proportion of back claims and sick duration (Gross et al. 2010). In contrast, Waddell et al. (2007) reported an 11% downward trend in the number of people who stayed off work.

*Frequency of doctor visits and pain relief medication use following the campaigns*: Buchbinder et al. (2008) reported a significant 15% – 20% reduction in the frequency of doctor visits related to back pain, but remained silent on the use of pain relief medication. Similarly, other campaigns were silent on the frequency of pain relief medication use and did not report the frequency of doctor visits (Gross et al. 2010; Suman et al. 2017; Waddell et al. 2007; Werner et al. 2007). However, even though no figures were given, Werner et al. (2007) highlight that they observed an increase in the number of surgery rates in both intervention and control counties, but observed no increase in referrals for imaging examination in the intervention county compared to the control.

*Work disability outcomes and effects of advice to stay active following the campaigns*: In all the campaigns (Buchbinder et al. 2008; Gross et al. 2010; Suman et al. 2017; Waddell et al. 2007; Werner et al. 2007), participants agreed and supported to stay and remain active regardless of back pain. Furthermore, significant shifts in back pain beliefs about staying active among the general population in Canada (*p* = 0.001), Scotland (*p* < 0.001) and Australia (OR 1.9–3.3) remained sustained for the duration of the studies (Buchbinder et al. 2008; Gross et al. 2010; Suman et al. 2017; Waddell et al. 2007).

## Discussion

This review reports on back pain community-based mass media campaigns. The campaigns included in this review aimed to address misconceptions such the need for rest and activity avoidance when experiencing back pain (Deneen et al. 2017). The campaigns reviewed included messages about physical, psychological, educational and work-related information to address pain, disability and work outcomes (Buchbinder et al. 2018; Werner et al. 2007). The campaign messages were aimed at promoting positive beliefs on back pain, encouraging self-coping strategies and functional activity. The purpose of the campaign messages was to encourage self-care and ownership of healthcare in individuals suffering from back pain.

Four campaigns that assessed the effectiveness of the ‘stay as active as possible’ message reported a statistically significant positive change. This finding implies that significantly more people were aware that they need to stay as active as possible if they have low back pain and that rest (especially bed rest) is not indicated. This is because rest slows down the natural progress of low back pain and influences work absenteeism (Hartvigsen et al. 2018). The increased awareness to stay as active as possible is therefore an important finding as it has spin-off effects on the prognosis and recovery period as well as financial implications at personal, institutional and national levels, as low back pain is one of the most common reasons for absenteeism (Buchbinder et al. 2018). Although this outcome was self-reported, a proxy measure to support behaviour change in the intervention groups could be reduced sick leave days or claims. Two of the campaigns (Buchbinder, Jolley & Wyatt 2001; Werner et al. 2007) indicated a reduction in sick leave and claims after the campaigns. These campaigns were conducted in HICs, but this message could be critical for LMICs where the belief to rest during low back pain episodes may be widespread among the general population.

Healthcare utilisation also reduced in the four campaigns that measured this outcome (Buchbinder et al. 2008; Gross et al. 2010; Waddell et al. 2007; Werner et al. 2007). Although this outcome was only statistically significant in the Australian campaign, all other campaigns showed a positive trend with respect to healthcare utilisation. The Australian campaign was more comprehensive than the campaigns in the other countries. For instance, they used prime time (on television and radio) to communicate their key messages. In addition, they included well-known personalities to deliver the campaign messages. However, it seems that even less expensive campaigns (Gross et al. 2010; Waddell et al. 2007; Werner et al. 2007) also had a positive effect on healthcare utilisation and the messages seem to have a positive effect on health seeking behaviours in people who experienced back pain. This is a pertinent finding for LMICs which may not have the resources for very expensive campaigns and have limited healthcare budgets. The message to stay active while experiencing back pain should therefore be considered for planned campaigns in lower resources settings where inefficient healthcare utilisation cannot be afforded.

The reduction in healthcare utilisation because of the back pain campaigns could be amplified by the reduced referral for X-rays shown in the Australia and Scotland campaigns (Buchbinder et al. 2001; Werner et al. 2007) which delivered messages that reduced the focus on spinal abnormalities and X-rays that rarely showing the reason for back pain. This is also indicated in the Lancet series, which highlight that liberal use of imaging does not reduce back pain disability or its long-term consequences. Instead, it triggers additional medical care costs and increases the risk of adverse outcomes, such as absence from work (Hoy et al. 2010). Recovery from back pain is aided by remaining active. Therefore, it is important to align practice with this evidence and especially for LMICs where imaging referral rates may still be high among patients with back pain.

One of the campaigns also reported on process evaluation (Suman et al. 2017). This campaign indicated that evaluation tested the cost-effectiveness and implementation strategy for the campaign. This suggests that process evaluation should be an important initial step when planning similar campaigns as it will assess the feasibility of recruitment, understanding and validity of the selected outcomes of the campaign. Process evaluation is particularly advisable for lower resource countries and regions where little is known about back pain beliefs, healthcare utilisation for back pain and management of back pain. A process evaluation also enables researchers to assess the feasibility of a campaign including barriers and facilitators before launching a more expensive interventional approach.

The campaigns were administered to the general population, and the interventions were clearly defined in all articles. Clearly defined interventions and populations are a good and helpful reference for future and similar research activities. Unfortunately, data outcomes for articles included in this review were not entirely comparable. This is because their focus, messages and data analysis and characteristics were differently done. In addition, there were missing data reports that made comparison and narration of the outcomes very difficult and is also a source of challenges for future research activities especially for resource-constrained areas. The number of articles available on back campaigns is very limited. This, to a great extent, may have impacted and influenced the findings and interpretations for this review. Similar campaigns are therefore recommendable especially in LMICs where these campaigns have not yet been done and the challenge of back pain is projected to increase in the next decade (Hoy et al. 2010).

## Conclusion

The review findings show that the back pain campaign message ‘stay as active as possible’ increased participants’ awareness to stay active and influenced positively their health beliefs and healthcare utilisation behaviours. The ‘stay as active as possible’ message is simple and easy to follow, which demonstrates that well-designed and simple messages have the potential to influence and promote health behaviour change in populations. The back campaigns were conducted in the general population in HICs. Even though their contents and methods are transferable to developing countries and populations frequently affected by back pain, their implementation must be tailored, and efficient; and cost-effective methods need to be explored. This is because back pain campaigns are seemingly an effective method in promoting back care and changing sickness behaviours and beliefs among affected individuals. Furthermore, over time, substantial and logical changes in back pain beliefs may lead to reduced fear and subsequently better self-coping for individuals during back pain episodes.

## References

[CIT0001] Buchbinder R., Gross D., Werner E.L. & Hayden J., 2008, ‘Understanding the characteristics of effective public health interventions for back pain and methodological challenges in evaluating their effects’, *Spine* 33, 74–80. 10.1186/1471-2474-9-112 18091029

[CIT0002] Buchbinder R., Jolley D. & Wyatt M., 2001, ‘Volvo award winner in clinical studies: Effects of a media campaign on back pain beliefs and its potential influence on management of low back pain in general practice’, *Spine* 26, 2535–2542. 10.1097/00007632-200112010-00005 11725233

[CIT0003] Buchbinder R., van Tulder M., Öberg B., Costa L.M., Woolf A., Schoene M. et al., 2018, ‘Low back pain: A call for action’, *Lancet* 391(10137), 2384–2388. 10.1016/S0140-6736(18)30488-4 29573871

[CIT0004] Forster N.E., Anema J.R., Cherkin D., Chou R., Cohen S.P., Gross D.P. et al., 2018, ‘Prevention and treatment of low back pain: Evidence, challenges and promising directions’, *Arthritis Research UK Primary Care Centre*, viewed 21 March 2018, from 10.1016/S0140-6736(18)30489-6 29573872

[CIT0005] Freimann T., Merisalu E. & Pääsuke M., 2015, ‘Effects of a home-exercise therapy programme on cervical and lumbar range of motion among nurses with neck and lower back pain: A quasi-experimental study’, *BMC Sports Science, Medicine and Rehabilitation* 7, 31 10.1186/s13102-015-0025-6 PMC4670527 26640694

[CIT0006] Gross D.P., Battie M.C., Wadelle G. & Buchbinder R., 2010, ‘Evaluation of a Canadian Back Pain Mass Media Campaign’, *SPINE* 35(8), 906–913, Lippincott Williams & Wilkins 10.1097/BRS.0b013e3181c91140 20308943

[CIT0007] Grotle M., Brox J.I., Veierød M.B., Glomsrød B., Lønn J.H. & Vøllestad N.K., 2005, ‘Clinical course and prognostic factors in acute low back pain: Patients consulting primary care for the first time’, *Spine* 30(8), 976e82. PMID: 10.1097/01.brs.0000158972.34102.6f 15834343

[CIT0008] Hartvigsen J., Natvig B. & Ferreira M., 2018, ‘Is it all about a pain in the back?’, *Best Practice and Research. Clinical Rheumatology* 27(5), 613–623. 10.1016/j.berh.2013.09.008 24315143

[CIT0009] Higgins J., Sterne J., Savović J., Page M., Hróbjartsson A., Boutron I. et al., 2016, ‘A revised tool for assessing risk of bias in randomized trials (RoB v2.0)’, *In Cochrane Methods* Cochrane Database of Systematic Reviews 10(Suppl 1), 29–31. 10.1002/14651858CD201601

[CIT0010] Higgins J.P.T., Green S., Chandler J.M.J., Boutron I. & Welch V., 2011, *Cochrane Handbook for systematic reviews of interventions version 5.1.0*, The Cochrane Collaboration, viewed n.d., from http://.cochrane.org.

[CIT0011] Hoy D., March L., Brooks P., Woolf A., Blyth F., Vos T. et al., 2010, ‘Measuring the global burden of low back pain’, *Best Practice and Research. Clinical Rheumatology* 24(2), 155–65. 10.1016/j.berh.2009.11.002 20227638

[CIT0012] Hoy D.G., Brooks P., Blyth F. & Buchbinder R., 2010, ‘The epidemiology of low back pain’, *Best Practice Research. Clinical Rheumatology* 24(6), 769–781. 10.1016/j.berh.2010.10.002 21665125

[CIT0013] Jaromi M.A., Kranicz J., Laczko T. & Betlehem J., 2012, ‘Treatment and ergonomics training of work-related lower back pain and body posture problems for nurses’, *Journal of Clinical Nursing* 21(11–12), 1776–1784. 10.1111/j.1365-2702.2012.04089.x 22594388

[CIT0014] Michaleff Z.A., Kamper S.J., Maher C.G., Evans R., Broderick C. & Henschke N., 2014, ‘Low back pain in children and adolescents: A systematic review and meta-analysis evaluating the effectiveness of conservative interventions’, *European Spine Journal* 23, 2046–2058. 10.1016/j.bjpt.2017.05.009 25070788

[CIT0015] Montegomery W., Sato M., Nagasaka Y. & Vietri J., 2017, ‘The economic and humanistic costs of chronic low back pain in Japan’, *Clinical Ergonomics and Outcomes Research* 9, 361–337. 10.2147/CEOR.S134130 PMC5491576 28694702

[CIT0016] Morris L.D., Daniels K.J., Ganguli B. & Louw Q.A., 2018, ‘An update on the prevalence of low back pain in Africa: A systematic review and meta-analysis’, *BMC Musculoskeletal Disorders* 19, 196 10.1186/s12891-081-2075-x 30037323 PMC6055346

[CIT0017] Nkhata L.A., Esterhuizen T.M., Siziya S., Phiri P.D.C., Nkandu E.M. & Shula H., 2015, ‘The prevalence and perceived contributing factors for work-related musculoskeletal disorders among nurses at the University Teaching Hospital in Lusaka, Zambia’, *Science Journal of Public Health* 3(4), 508–513. 10.11648/j.sjph.20150304.18

[CIT0018] Qaseem A., Wilt T.J., McLean R.M. & Forciea M.A., 2017, ‘Clinical guidelines committee of the American College of Physicians. Noninvasive treatments for acute, subacute, and chronic low back pain: A clinical practice guideline from the American College of Physicians’, *Annals of Internal Medicine* 166, 514–530. 10.7326/M16-2367 28192789

[CIT0019] Roland M., Waddell G., Moffat J.K., Burton K., Main C. & Cantrell T., 1996, *The back book*, The Stationery Office, London 10.1136/bmj.38282.607859.AE

[CIT0020] Soon L.K. & Jong E.L., 2010, ‘Development of an intervention to prevent work-related musculoskeletal disorders among hospital nurses based on the participatory approach’, *Applied Ergonomics* 14, 454–460. 10.1016/j.apergo.2009.09.007 19875100

[CIT0021] Sterne J.A.C., Hernán M.A., Reeves B.C., Savović J., Berkman N.D., Viswanathan M. et al., 2016, ‘ROBINS-I: A tool for assessing risk of bias in non-randomised studies of interventions’, *BMJ* 355, i4919 10.1136/bmj.i4919 27733354 PMC5062054

[CIT0022] Stochkendahl M.J., Kjaer P., Hartvigsen J., Kongsted A., Aaboe J., Andersen M. et al., 2018, ‘National clinical guidelines for non-surgical treatment of patients with recent onset low back pain or lumbar radiculopathy’, *European Spine Journal* 27(1), 60–75. 10.1007/s00586-017-5099-2 28429142

[CIT0023] Suman A., Frederieke G., Schaafsma L., Bamarni J., Maurits W., van Tulder J. et al., 2017, ‘A multimedia campaign to improve back beliefs in patients with non-specific low back pain: A process evaluation’, *BMC Musculoskeletal Disorders* 18, 200 10.1186/s12891-017-1551-z 28521761 PMC5437407

[CIT0024] Thompson H., Craig P., Hilton-Boon M., Campbell M. & Katikiredd S.V., 2018, ‘Applying the ROBINS-I tool to natural experiments: An example from public health’, *Systematic Reviews* 7(1), 15 10.1186/s13643-017-0659-4 29368630 PMC5784724

[CIT0025] Tufanaru C., Moola S., Munn Z., Sears K., Sfetcu R. Qureshi R., et al., 2015, ‘Conducting systematic reviews of association: The Joanna Briggs Institute’s approach’, *International Journal of Evidence-Based Healthcare* 13(3), 163–169. 10.1097/XEB.0000000000000064 26262566

[CIT0026] Verhagen A.P., De Vet H.C., Die Bie R., Kessels A., Broers M., Bouer L. et al., 1998, *The Pedro scale*, viewed 19 January 2018, from http://staff.unak.is/andy/NursResearchMethods0506/Pedro/PEDroscale.doc.

[CIT0027] Vos T., Hoy D., Bain C., William G., March L., Brook P. et al., 2012, ‘A systematic review of global prevalence of low back pain’, *Arthritis and Rheumatology* 64(6), 2028–2037. 10.1002/art.34347 22231424

[CIT0028] Waddell G., O’Connor M., Boormans S. & Torsney B., 2007, ‘Working backs. A public and professional health education campaign for back pain’, *Spine* 32, 2139–2143. 10.1097/BRS.0b013e31814541bc 17762817

[CIT0029] Werner E.L., Ihlebaek C., Laerum E., Wormgoor M.E.A. & Indahl A., 2007, ‘The effect of media campaign on popular beliefs about LBP and eventual changes in sick leave, imaging examination’, *Patient Education and Counseling* 71(2), 198–203. 10.1016/j.pec.2007.12.009 18242932

